# Sensory Lexicon Construction and Quantitative Descriptive Analysis of Jiang‐Flavor Baijiu

**DOI:** 10.1002/fsn3.4652

**Published:** 2025-02-18

**Authors:** Junjie Niu, Yubo Yang, Lei Zhao, Nian Cao, Xiaolin Xiong, Zhenyu Yun, Fan Yang, Yang Xu, Huabin Tu, Kui Zhong, Houyin Wang, Haiyan Gao, Yao Zhang, Zhen Qin, Li Wang, Bolin Shi

**Affiliations:** ^1^ School of Life Sciences Shanghai University Shanghai China; ^2^ Maotai Distillery Co. Ltd. Renhuai China; ^3^ Institute of Agri‐food Standardization China National Institute of Standardization Beijing China; ^4^ Key Laboratory of Food Sensory Analysis State Administration for Market Regulation Beijing China

**Keywords:** flavor wheel, Jiang‐flavor Baijiu, quantitative descriptive analysis, sensory characteristics, sensory lexicon

## Abstract

Jiang‐flavor Baijiu (JFB) is one of the most complex and representative types of traditional Chinese liquor, known for its unique flavor, which is popular among Chinese consumers. Previous studies primarily focused on detecting and identifying volatile compounds but lacked comprehensive descriptions and precise differentiation of their sensory qualities. To explore the sensory characteristics of JFB, this study collected widely recognized sensory descriptors from literature, standards, free descriptive questionnaires, and Pivot Profile experiments, which were then textually analyzed and merged to yield 88 sensory descriptors. M‐value and multivariate statistical methods were then used to narrow these down to 35 sensory descriptors, for which corresponding definitions and physical references were established, forming a sensory lexicon for JFB. A quantitative descriptive analysis (QDA) was performed on JFB samples using the sensory lexicon. To ensure the reliability of the analysis, the panel's performance was also evaluated. Hierarchical cluster analysis (HCA), radar plots, and one‐way analysis of variance (ANOVA) demonstrated that the established sensory lexicon accurately reflects the sensory profile of JFB and can be used to differentiate the sensory characteristics of samples from different price ranges or regions. This study opens new avenues for the standardization of quantitative descriptive analysis for JFB and provides a case study for constructing sensory lexicons for other types of Baijiu in China.

## Graphical abstract

In conclusion, we carried out the construction of a sensory lexicon for JFB for the first time and conducted a series of analyses on samples, descriptors, and evaluation groups. The consistency of the evaluation panel was good, the sensory characteristics of the samples were distinguished, and the ability of the descriptors to distinguish was tested.

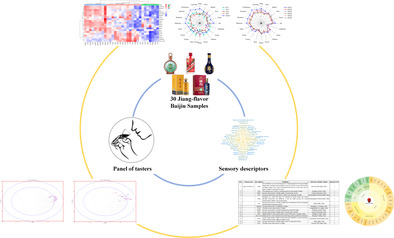

## 1 Introduction

**FIGURE 1:**
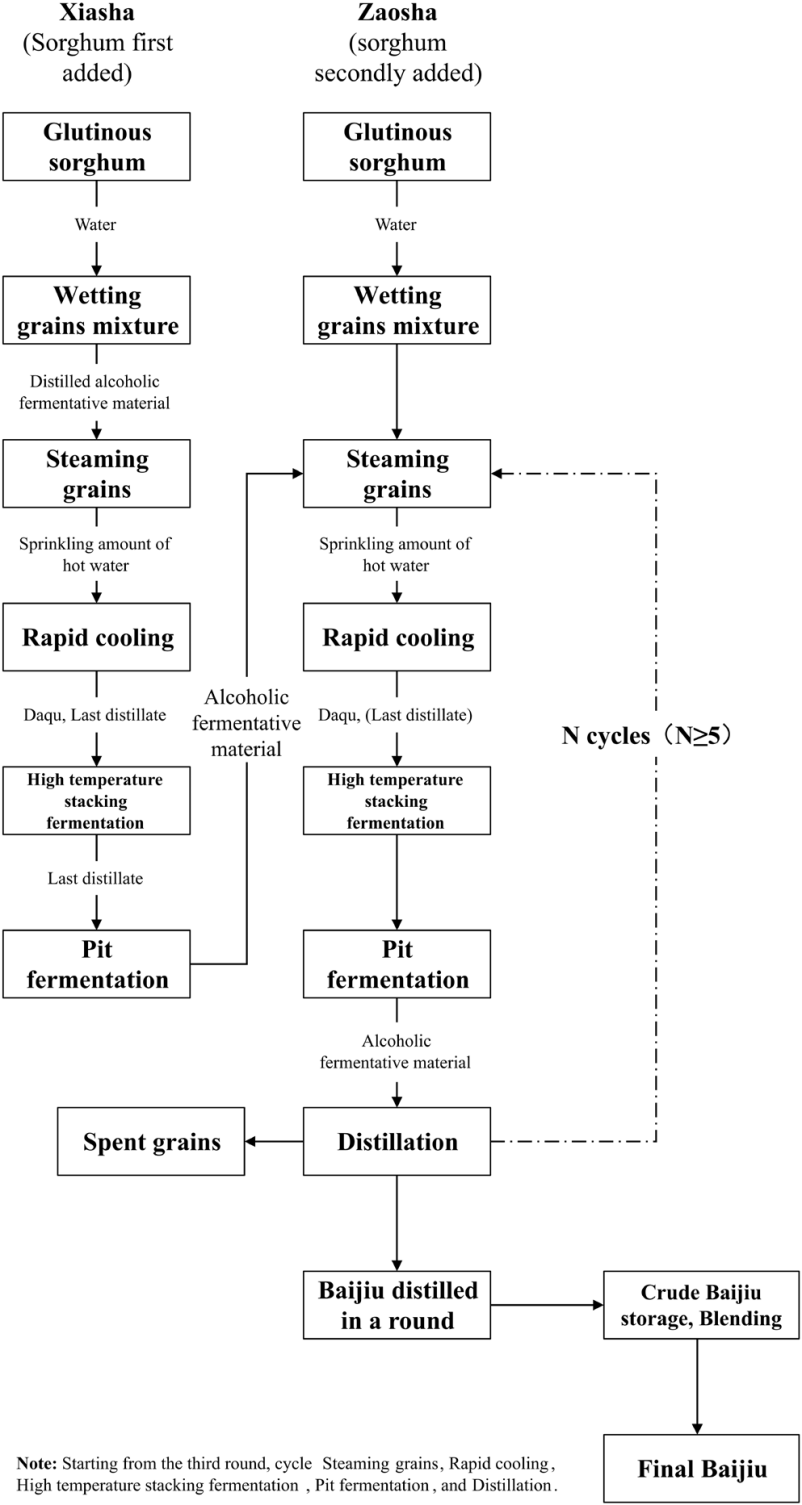
Jiang‐flavor Baijiu production process.

Baijiu, the national liquor of China, has a history of over 2000 years and is one of the world's six major distilled spirits, widely cherished by Chinese consumers (Wu et al. 2022; Yan, Zhang, et al. 2021). Baijiu's rich flavor compounds and unique sensory experience have placed it high on global spirit consumption lists, making it a symbolic alcoholic beverage with a prestigious reputation in Eastern cultures (Wang et al. 2021; Li, Zhang, and Sun 2023). Jiang‐flavor Baijiu (JFB), also known as Sauce flavor Baijiu (Ge et al. 2023) or Moutai flavor Baijiu (Yan, Zhang, et al. 2021), is one of the 12 Baijiu flavor types and is considered the most mysterious due to its complex and rich chemical composition (Xue et al. 2023). JFB has a complex production process and an extended production cycle, involving two sorghum additions, nine steamings, eight fermentations, and seven distillations within a 1‐year cycle (Figure 1). The base wine, produced through the “12,987” process with varying rounds and flavor characteristics, is then stored and blended to create the final Baijiu (Huang et al. 2023; Niu et al. 2024). The main production areas of JFB are Guizhou and Sichuan provinces, both located in the Chishui River basin (Qin et al. 2023). Quality determines value, and flavor is a key aspect of Baijiu's quality and evaluation. Aroma, in particular, is a crucial indicator of JFB's quality (Li, Zhang, Gao, et al. 2023; Niu, Zhao, et al. 2023; Wang et al. 2020). Whether for quality control of the final Baijiu or for grading and blending the base wine, professional tasters rely on their sensory evaluation of samples. The current technical system of Baijiu tasting mainly originates from the five National Wine Tasting Conferences (1952–1989), where the methods and steps of Baijiu tasting, along with sensory standards and terminology for each type of Baijiu, were established. The selection and assessment mechanisms for professional tasters were established through standards and qualification exams that continue to this day (Niu, Shi, et al. 2023). JFB has a unique flavor and complex aroma profile. Research on its taste and mouthfeel primarily relies on sensory evaluation by professional tasters (trained panelists) in distilleries, while aroma research currently focuses on detecting volatile compounds using modern instrumental techniques, supplemented by sensory evaluation from expert panels (Wu et al. 2022). However, instrumental detection of aroma substances, such as gas chromatography–mass spectrometry, does not fully capture people's perception of JFB, highlighting the need for reliable methods to describe it.

Sensory quality can be assessed using both analytical methods (objective), and consumer hedonic research (subjective) (García‐Gómez et al. 2022). A sensory lexicon is a standardized vocabulary that objectively describes the sensory properties of foods, developed based on scientific principles and standardized procedures, allowing different groups to consistently describe foods (Lawless and Civille 2013). A sensory lexicon is valuable for sensory researchers, producers, and consumers to understand the sensory properties of products. It has been widely used in the sensory evaluation of various food products, such as coffee (Chambers IV et al. 2016), cheese (Gulzar et al. 2020), wine (Souza Gonzaga et al. 2019), chocolate (De Pelsmaeker et al. 2019) and Sufu (Chen and Chung 2016). Quantitative Descriptive Analysis (QDA) is commonly used to describe sensory characteristics such as appearance, aroma, texture, taste, mouthfeel, and aftertaste, making it a highly detailed and effective method (Solomando et al. 2021). The sensory information obtained from QDA can also help food companies make more accurate business decisions (Wang et al. 2023). To our knowledge, most JFB sensory evaluations in distilleries are currently conducted in the form of grading, which is a comprehensive assessment but lacks the precision and digitalization of QDA. QDA has been used in some JFB sensory studies, but the sensory descriptors (sensory lexicon) were often discussed only by the panel, leading to issues such as incomplete descriptors and the absence of standardized references for attribute intensity. Additionally, the lack of definitions for descriptive terms further complicates the issue of standardization. This makes it unsuitable for communication between different panels and for multiple applications of the sensory lexicon.

In this study, 30 Jiang‐flavor Baijiu (JFB) samples from the Chinese market were evaluated to establish an objective sensory evaluation method and to accurately describe the sensory attributes of JFB. The objectives of this study were to (a) develop a sensory lexicon with definitions and reference standards to explain the sensory attributes of JFB; (b) evaluate the sensory characteristics of 30 JFB samples using the QDA method based on the sensory lexicon; and (c) verify the effectiveness of the descriptors and the QDA method in evaluating the sensory characteristics of JFB.

## 2 Materials and Methods

### 2.1 Samples

In the Chinese market, 30 samples of mainstream Jiang‐flavor Baijiu (JFB) were selected, considering factors such as price, brand, and production region (Table 1). After purchase, the samples were stored at room temperature in their original, unopened packaging. The samples were unpacked and labeled with a three‐digit random code within 1 h before the tasting. Following the requirements of GB/T 10345‐2022 (2017). *Method of Analysis for Baijiu*, the JFB samples were poured into clean, dry standard tasting glasses with a footed base, in volumes of 15–20 mL.

**TABLE 1:** Price, production region, and area information for 30 JFBs.

Code	Price	Production region	Area
SC1	H1	SCCQ	Shehong City, Sichuan Province
SC2	H1	SCCQ	Gulin County, Sichuan Province
GZ1	H1	GZCQ	Jinsha County, Guizhou Province
MT1	H1	MTCQ2	Maotai Town, Guizhou Province
MT01	H1	MTCQ1	Maotai Town, Guizhou Province
MT2	H2	MTCQ2	Maotai Town, Guizhou Province
XS1	H2	XSCQ	Xishui County, Guizhou Province
XS2	H2	XSCQ	Xishui County, Guizhou Province
MT02	H2	MTCQ1	Maotai Town, Guizhou Province
SD1	H2	SDCQ	Dezhou City, Shandong Province
MT3	M1	MTCQ2	Maotai Town, Guizhou Province
HN1	M1	HNCQ	Changde City, Hunan Province
SC3	M1	SCCQ	Yibin City, Sichuan Province
XS3	M1	XSCQ	Xishui County, Guizhou Province
MT4	M1	MTCQ2	Maotai Town, Guizhou Province
GZ2	M2	GZCQ	Guiyang City, Guizhou Province
MT5	M2	MTCQ2	Maotai Town, Guizhou Province
GZ3	M2	GZCQ	Zunyi City, Guizhou Province
MT03	M2	MTCQ1	Maotai Town, Guizhou Province
RH1	M2	RHCQ	Renhuai City, Guizhou Province
MT04	L1	MTCQ1	Maotai Town, Guizhou Province
HHL10	L1	SCCQ	Gulin County, Sichuan Province
MT6	L1	MTCQ2	Maotai Town, Guizhou Province
MT05	L1	MTCQ1	Maotai Town, Guizhou Province
MT06	L1	MTCQ1	Maotai Town, Guizhou Province
SC5	L2	SCCQ	Yibin City, Sichuan Province
XS4	L2	XSCQ	Xishui County, Guizhou Province
MT7	L2	MTCQ2	Maotai Town, Guizhou Province
RH2	L2	RHCQ	Renhuai City, Guizhou Province
RH3	L2	RHCQ	Renhuai City, Guizhou Province

*Note:* (1) Price: H1 samples > 1500 yuan (RMB), H2 samples 1000–1500, M1 samples 700–1000, M2 samples 500–700, L1 samples 300–500, L2 samples < 300. (2) Production region: MTCQ1, Maotai production region 1, for the core production area of Maotai Town, Guizhou Province; MTCQ2, Maotai production region 2, the rest of the area outside the core production region of Maotai town.

Abbreviations: GZCQ, Guizhou production region, the rest of the geographical area in Guizhou province except the above‐mentioned area; HNCQ, Hunan production region; RHCQ, Renhuai production region, produced in Renhuai city, Guizhou Province; SCCQ, Sichuan production area, which refers to Shehong city, Gulin county, and Yibin city in this study; SDCQ, Shandong production region; XSCQ, Xishui production region, produced in Xishui county, Guizhou Province.

### 2.2 Panel

The panel included 4 female and 19 male participants, aged between 26 and 46. All participants were certified tasters, holding certificates from the China Alcoholic Drinks Association (CADA) or the China National Food Industry Association (CNFIA). They were core members of the enterprise's Quality Department, Wine Design Center, Technology Center, and Wine Making Workshop, where their daily work involved tasting various Jiang‐flavor Baijiu (JFB) based on aroma, taste, and other sensory attributes. Their experience in Baijiu tasting ranged from 2 to 27 years. Among them were 5 national Baijiu judges and 1 provincial Baijiu judge. For the sensory lexicon construction, 11 members formed Panel 1 and participated in the tasting experiment, while for the QDA part, 13 members formed Panel 2. Before conducting the experiments, both panels underwent a week of appropriate training. The study was reviewed and approved by the Human Study Ethics Committee of Beijing Forestry University and informed consent was obtained from each participant prior to their participation in the study.

### 2.3 Tasting Organization

A balanced design was implemented on 30 samples, divided into 5/6 sessions for each of the two tasting experiments, with 6/5 samples evaluated in each session. Each session lasted 1 h, with a 15‐min rest and adjustment period between sessions, during which pure water was used for mouth cleansing. The tasting intensity in both experiments was lower than that of the tasters' daily evaluations (4 sessions of 5 samples each within half a day). The experiments took place in a tasting room that met the light, temperature, relative humidity, and air quality standards specified in *GB/T 10345–2022. Method of Analysis for Baijiu*. The panelists evaluated the samples' aroma, taste, mouthfeel, residual taste, and aroma in an empty cup in sequence, following the standard rules and techniques used in daily Baijiu evaluations.

### 2.4 Sensory Lexicon Generation and Screening

In this study, sensory descriptors of Jiang‐flavor Baijiu (JFB) were collected through multiple methods: 684 descriptors were gathered and extracted from literature and standards, 1661 descriptors were obtained from free descriptor questionnaires, and 4005 descriptors were generated using the Pivot Profile method. After textual analysis, these descriptors were sorted and consolidated into a comprehensive list of 88 JFB sensory descriptors, which include 36 types of aroma, 9 aroma characteristics, 5 taste descriptors, 6 mouthfeel descriptors, 4 residual taste descriptors, and 28 types of aroma in an empty cup.

In accordance with GB/T 16861‐1997 (1997). *Sensory Analysis‐Identification and Selection of Descriptors for Establishing a Sensory Profile by a Multidimensional Approach*, panel 1 was assembled to evaluate 30 JFB samples and score each sensory attribute using a 5‐point scale. The collected data were then analyzed using multivariate statistical methods, including the geometric mean (*M*), hierarchical cluster analysis (HCA), and principal component analysis (PCA), to gradually filter out representative sensory descriptors of JFB that could highlight differences between products. These descriptors were visually represented in the form of a flavor wheel.

### 2.5 Quantitative Descriptive Analysis (QDA) for Jiang‐Flavor Baijiu

With reference to the Baijiu sensory descriptors defined in GB/T 33405‐2016 (2016). *Terminology of Baijiu Sensory Evaluation*, 30 JFB samples were prepared. Tasters were then organized for discussions and tastings to establish definitions for the screened sensory descriptors and to identify corresponding physical reference samples. This process resulted in the creation of a JFB sensory lexicon (Table 2). Using this lexicon as a foundation, Panel 2 was assembled to conduct Quantitative Descriptive Analysis (QDA) on 30 samples, scoring each on a 5‐point scale.

**TABLE 2:** Jiang‐flavor Baijiu sensory lexicon.

NO.	Category (no.)	Descriptors	Definition	Reference (brand): volume	Intensity (0–5)
1	Types of aroma (15)	Jiang	The traditional soy sauce brewing process of high‐temperature brewing and high‐temperature stacking and fermentation is used to make the aroma characteristics of Baijiu; similar to the aroma of fermented foods such as sweet sauce and soy sauce; similar to sauce, and soy sauce	Jiaocang 1988 (Xijiu), 20 mL	3
2		Chen	The aging process results in a naturally matured aroma profile of Baijiu	Hanjiang (Moutai), 20 mL	3
3		Baked	Aroma characteristics of Baijiu similar to roasted grain cereals	Liudao (Gubeichun), 20 mL	3
4		Sour	Aroma characteristics presented by volatile acid components in Baijiu	RenshuaiqiaoB (Moutai health), 20 mL	3
5		Grain	Sorghum, rice, wheat, and other raw materials of various grains and cereals through fermentation and distillation, so that the Baijiu presents the aroma characteristics similar to the steamed grain	RenshuaiqiaoB (Moutai health), 20 mL	3
6		Qu	Aroma characteristics of Baijiu resulting from fermentation with the participation of daqu(Sugar fermentation agent)	Junpin (Xijiu), 20 mL	3
7		Aldehyde	Aroma characteristics of aldehydes in Baijiu	Shangjiang 15 (Wuling), 20 mL	3
8		Fruity	Fruit‐like aroma characteristics of Baijiu	Honghualang 10 (Langjiu), 20 mL	3
9		Ethanol	Aroma characteristics of alcohol components presented in Baijiu	Hanjiang (Moutai), 20 mL	3
10		Sweet	Aroma characteristics of Baijiu with a sweetness‐like sensation	Liudao (Gubeichun), 20 mL	3
11		Burnt	Baijiu exhibits an odor profile similar to that of burnt paste of organic matter	Zhenling 9 (Guihe), 20 mL	3
12		Raw wood	Baijiu present a more harmonious and refreshing aromatic profile than Aldehyde	Zhenling 9 (Guihe), 20 mL	3
13		Grassy	Aroma characteristics similar to fresh fruits and vegetables or greens and grass, with a distinctly unripe, slightly astringent aroma	Maotaichun (Moutai health), 20 mL	3
14		Floral	Aroma characteristics of Baijiu similar to that of a plant flower/flora	Junpin (Xijiu), 20 mL	3
15		Nutty	Nut‐like aroma characteristics of Baijiu	Jiaocang 1988 (Xijiu), 20 mL	3
16	Features of aroma (5)	Harmony	Appropriateness of the combination of aroma types	Honghualang 10 (Langjiu), 20 mL	3
17		Richness	Degree of each type of aroma in the overall aroma	Liudao (Gubeichun), 20 mL	3
18		Intensity	Degree of concentration of the overall aroma	Shangjiang 15 (Wuling), 20 mL	3
19		Cleanliness	Cleanliness of the overall aroma, with or without off‐odor	RenshuaiqiaoB (Moutai health), 20 mL	3
20		Layering	Degree to which each aroma type is represented in turn according to time or concentration	Liudao (Gubeichun), 20 mL	3
21	Taste (3)	Acid_T	Vinegar‐like taste characteristics of certain organic acids in Baijiu	Hanjiang (Moutai), 20 mL	3
22		Sweet_T	Sucrose‐like taste characteristics of certain substances (e.g., polyols) in Baijiu	Maotaichun (Moutai health), 20 mL	3
23		Bitter_T	Bitter almond‐like taste characteristics of certain substances in Baijiu	Zhenling 9 (Guihe), 20 mL	3
24	Mouthfeel (4)	Harmony_M	The comfort level of the various sensory combinations of Baijiu in the mouth	Liudao (Gubeichun), 20 mL	3
25		Fullness_M	The richness of the various sensations of Baijiu in the mouth	Liudao (Gubeichun), 20 mL	3
26		Softness_M	Degree of smoothness and harshness in the mouth of Baijiu	Jiaocang 1988 (Xijiu), 20 mL	3
27		Cleanliness_M	The degree of lubrication and cleanliness felt when the Baijiu is swallowed	Guotai 15 (Guotai), 20 mL	3
28	Residual taste (2)	Persistance_RT	Duration of the combined olfactory and gustatory sensations produced by the swallowing of Baijiu	Zhenling 9 (Guihe), 20 mL	3
29		Cleanliness_RT	Cleanliness of the mouth after swallowing Baijiu, with or without off‐flavors	Maotaichun (Moutai health), 20 mL	3
30	Aroma in empty cup (6)	Jiang_EC	When an empty cup of Baijiu is left for a period of time, an aroma similar to that of fermented foods such as sweet sauce and soy sauce is detected	RenshuaiqiaoB (Moutai health), 20 mL	3
31		Qu_EC	When an empty cup of Baijiu is left for a period of time, aroma characteristics of the Baijiu presented by the participation of fermentation such as Daqu, Branqu, or Xiaoqu, etc. can be smelled	Liudao (Gubeichun), 20 mL	3
32		Sour_EC	When an empty cup of Baijiu is left for a period of time, an aroma similar to that of volatile acids is detected on the nose	Hanjiang (Moutai), 20 mL	3
33		Baked_EC	When an empty cup of Baijiu is left for a period of time, an aroma similar to that of roasted grain is detected	RenshuaiqiaoB (Moutai health), 20 mL	3
34		Chen_EC	When an empty cup of Baijiu is left for a period of time, smell the aroma characteristics of Baijiu naturally formed by aging	Gui 15 (Guijiu), 20 mL	3
35		Persistance_EC	The persistence of the aroma on the nose of an empty cup filled with Baijiu after a period of time	Liudao (Gubeichun), 20 mL	3

### 2.6 Data Analysis

One‐way analysis of variance (ANOVA) with Duncan's test (*p* ≤ 0.05) was conducted using SPSS Statistics 25 software. The panel's performance was assessed with PanelCheck (Version 1.4.2, http://www.panelcheck.com). Principal component analysis (PCA) and heatmaps were generated via an online platform (https://www.bioinformatics.com.cn). Hierarchical cluster analysis (HCA) and radar plots were created using Origin 2022 software. Additional data processing was carried out with Microsoft Office Excel 2021.

## 3 Results and Discussion

### 3.1 Construction and Screening of Descriptors

#### 3.1.1 Screening of Jiang‐Flavor Baijiu Sensory Profile Descriptors

The comprehensive search for descriptors through literature, standards, free description questionnaires, and Pivot Profile analysis (Niu et al. 2024; Yang et al. 2024) laid the groundwork for the quantitative sensory description of Jiang‐flavor Baijiu (JFB). Among the merged and screened descriptors (88 in total), several effectively describe the sensory characteristics of JFB. The *M*‐values of these descriptors (Table 3), which reflect their significance to the samples, were used to retain descriptors with values higher than the average for each category: Types of aroma, Features and style of aroma, Taste, Mouthfeel, Residual taste, and Aroma in empty cup (0.1544, 0.4769, 0.1679, 0.4624, 0.3167, and 0.1482, respectively). Specifically, 13 descriptors were retained for aroma type, 6 for aroma profile, 3 for taste, 4 for mouthfeel, 2 for residual taste, and 13 for aroma in empty cup. In the context of taste, esters contributed to Baijiu's sweet taste (Sun et al. 2021), acid taste was commonly identified in JFB (Li, Wang, Xu, et al. 2023), and bitter taste was noted as an off‐flavor, with ongoing research into its source (Xue et al. 2023). Regarding mouthfeel, indicators such as softness, fullness, harmony, and persistence were previously established for JFB base wines (Wu, Chen, et al. 2023), and in this study, “cleanliness” (in aroma, mouthfeel, and aftertaste) was used to describe the absence of off‐odors or off‐flavors like oily and rancid notes. While taste and mouthfeel descriptors were few but sufficient to capture JFB's sensory characteristics, the number of aroma descriptors was relatively high, making them less practical for guiding future JFB tastings. After panel discussions, tasters found that perceiving aroma in an empty cup was more challenging than direct smelling. Consequently, six aroma indicators—Sweet_EC, Fruity_EC, Grain_EC, Burnt_EC, Grassy_EC, and Aldehyde_EC—were removed, and the remaining aroma descriptors were categorized for PCA and HCA analysis.

**TABLE 3:** Geometric mean *M* of sensory descriptors of Jiang‐flavor Baijiu.

NO.	Descriptors	*M*	NO.	Descriptors	*M*	NO.	Descriptors	*M*
Types of aroma (36)			32	Honey	0.0397	60	Burnt_RT	0.1767
1	**Jiang**	0.6645	33	Herbal	0.0350	Aroma in empty cup (28)		
2	**Chen**	0.3237	34	Mild	0.0239	61	**Jiang_EC**	0.4758
3	**Baked**	0.3165	35	Creamy	0.0074	62	**Richness_EC**	0.4016
4	**Sour**	0.3083	36	Chocolate	0.0014	63	**Persistance_EC**	0.3198
5	**Grain**	0.2901	Features and style of aroma (9)			64	**Qu_EC**	0.2715
6	**Qu**	0.2896	37	**Harmony**	0.5884	65	**Sour_EC**	0.2199
7	**Aldehyde**	0.2796	38	**Richness**	0.5874	66	**Baked_EC**	0.2133
8	**Fruity**	0.2568	39	**Layering**	0.5434	67	**Chen_EC**	0.2122
9	**Ethanol**	0.2347	40	**Intensity**	0.5394	68	**Sweet_EC**	0.1954
10	**Sweet**	0.2211	41	**Persistence**	0.4978	69	**Fruity_EC**	0.1942
11	**Burnt**	0.1935	42	**Cleanliness**	0.4975	70	**Grain_EC**	0.1939
12	**Raw wood**	0.1687	43	Quiet and tastefully laid out	0.4639	71	**Burnt_EC**	0.1841
13	**Grassy**	0.1593	44	Softness	0.4431	72	**Grassy_EC**	0.1676
14	Roasted	0.1391	45	Dull feeling	0.1313	73	**Aldehyde_EC**	0.1585
15	Rancid	0.1313	Taste (5)			74	Quiet and tastefully laid out_EC	0.1444
16	Fermented mud	0.1221	46	**Acid_T**	0.3498	75	Roasted_EC	0.1065
17	Sesame	0.1171	47	**Sweet_T**	0.2385	76	Floral_EC	0.1006
18	Oily	0.1097	48	**Bitter_T**	0.2059	77	Off‐odor_EC	0.0895
19	Grassy	0.1091	49	Salty_T	0.0278	78	Fermented mud_EC	0.0824
20	Nutty	0.1063	50	Umani_T	0.0177	79	Rancid_EC	0.0789
21	Aroma of new Baijiu	0.1014	Mouthfeel (6)			80	Oily_EC	0.0752
22	Salted vegetable	0.1001	51	**Harmony_M**	0.5813	81	Musty_EC	0.0719
23	Floral	0.0953	52	**Fullness_M**	0.5672	82	Sesame_EC	0.0589
24	Musty	0.0953	53	**Softness_M**	0.5609	83	Rubbery_EC	0.0476
25	Crushed grain	0.0951	54	**Cleanliness_M**	0.5048	84	Jiao_EC	0.0334
26	Rubbery	0.0852	55	Layering_M	0.4587	85	Honey_EC	0.0168
27	Jiao	0.0843	56	Astringency	0.1015	86	Herbal_EC	0.0163
28	Off‐odor	0.0808	Residual taste (4)			87	Nutty_EC	0.0158
29	Distilled grain	0.0756	57	**Persistance_RT**	0.5134	88	Creamy_EC	0.0041
30	Material	0.0733	58	**CLeanliness_RT**	0.3823			
31	Woody	0.0578	59	Thin_RT	0.1945			

*Note:* (1) Bolded descriptors indicate that their *M*‐value is greater than the average of the *M*‐values of the descriptors in that dimension (types of aroma, 0.1544; Features and style of aroma, 0.4769; Taste, 0.1679; Mouthfeel, 0.4624; Residual taste, 0.3167; and Aroma in empty cup, 0.1482). (2) *M* is the geometric mean, *M* = $\sqrt{F*I}$. Where *F* was the percentage of the number of times the descriptor was actually addressed to the total number of times all possible addresses for that descriptor were addressed, and I was the percentage of the strength of a descriptor that was actually given by the panel to the maximum possible strength that could be obtained for that descriptor. (3) EC, empty cup; M, mouthfeel; T, taste; RT, residual taste.

**FIGURE 2:**
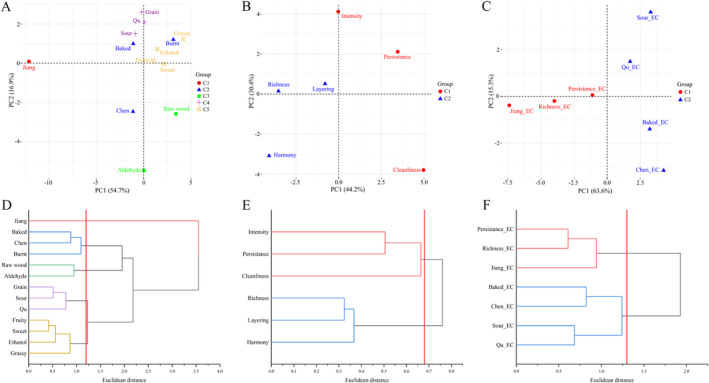
Principal component analysis (PCA) and hierarchical cluster analysis (HCA) of sensory descriptors of JFB. (A, D) Types of aroma, (B, E) features and style of aroma, (C, F) aroma in empty cup.

For the aroma type category, the first two principal components explained 71.6% of the variability, as shown in Figure 2A. This indicates that the 13 descriptors captured most of the variability within the JFB samples and were effective in describing the aroma types of JFB. The results of the HCA (Figure 2D) grouped the 13 descriptors into five categories, with one category representing Jiang alone. However, the panel concluded that each descriptor represented a distinct aroma type, and therefore, they could not be combined. Additionally, the panel noted that floral and nutty aromas, though less frequent, were significant in differentiating individual samples, suggesting their importance despite their low *M*‐values. These low *M*‐values likely resulted from their infrequent occurrence but high intensity when present (i.e., low *F*‐value and high *I*‐value). Consequently, the original 13 aroma types, along with floral and nutty, were selected as descriptors for the quantitative evaluation of JFB's aroma types.

Similarly, for aroma characteristics and empty cup aroma (Figure 2B,C), the cumulative variance contributions of the first two principal components were 74.6% and 78.9%, respectively, capturing most of the variability in the aroma characteristics and empty cup aroma of JFB samples. The HCA (Figure 2E,F) revealed that intensity and persistence were clustered together, indicating a strong correlation between aroma concentration (intensity) and how long the aroma lasted (persistence). As a result, persistence was removed, with its meaning internalized within intensity. Persistence_EC and Richness_EC were also clustered into one category, leading to the deletion of Richness_EC, which was internalized within the assessment of the amount and strength of each type of empty cup aroma (e.g., Jiang_EC, Qu_EC). Jiang_EC, Qu_EC, and Sour_EC have previously been used as evaluation indexes for Jiang‐flavor Baijiu's empty cup aroma (Qin et al. 2024). Additionally, definitions were established for these descriptors, along with physical references and intensity levels for the corresponding sensory attributes (Table 2).

#### 3.1.2 Construction of Jiang‐Flavor Baijiu Flavor Wheel

**FIGURE 3:**
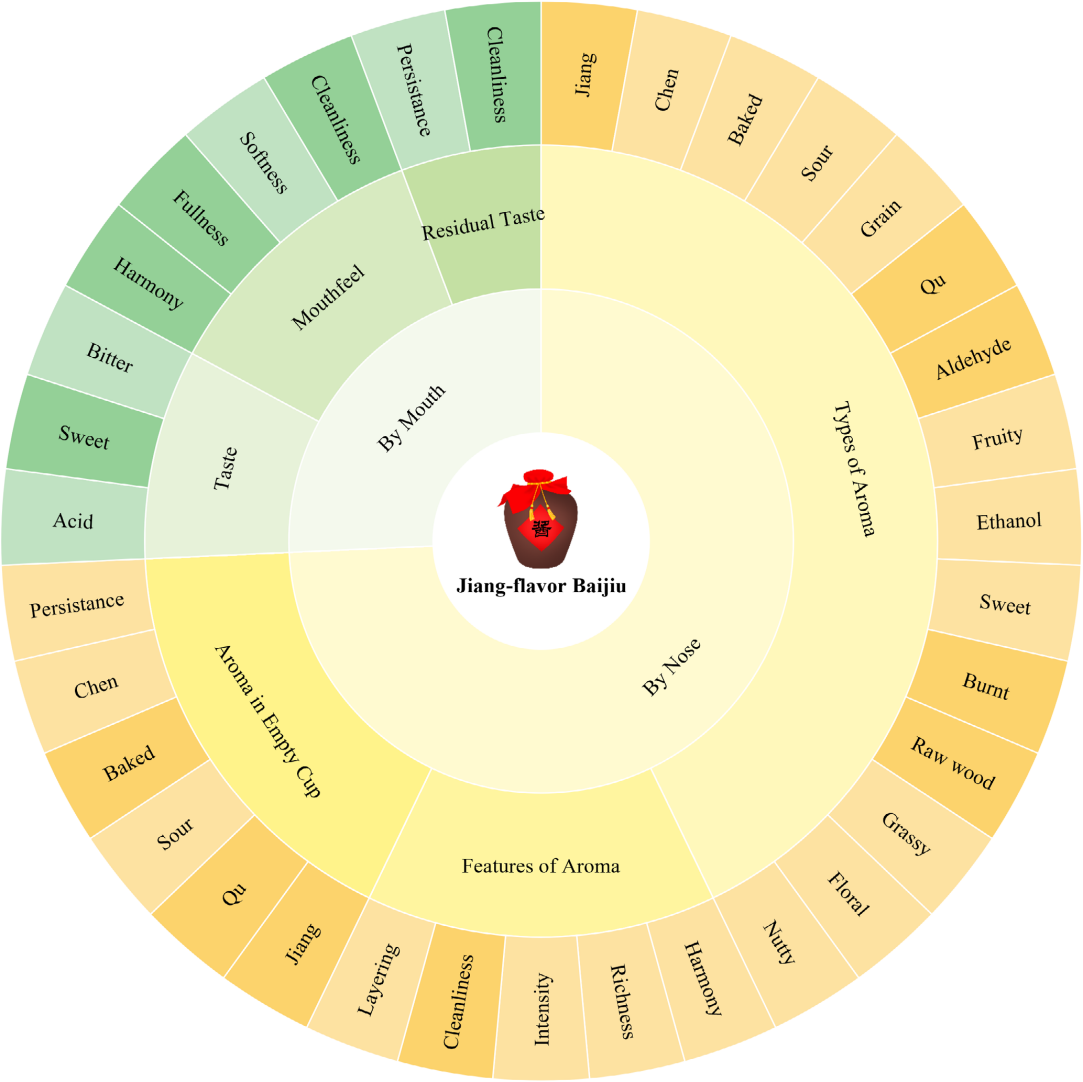
Flavor wheel of JFB based on multivariate statistical selection.

Thirty‐five sensory descriptors with strong descriptive and discriminative abilities, identified through *M*‐value, PCA, and HCA screening, were organized into a flavor wheel for JFB (Figure 3). The flavor wheel is divided into three levels, containing 2, 6, and 35 terms from the innermost to the outermost circle. The first level differentiates the sensory modes as “by nose” or “by mouth.” The second level includes six sensory dimensions: Types of Aroma, Features of Aroma, Aroma in Empty Cup, Taste, Mouthfeel, and Residual Taste. The third level comprises a sensory lexicon of 35 specific descriptors for JFB. This flavor wheel and sensory lexicon form the foundation for the subsequent QDA.

### 3.2 Results of the QDA

#### 3.2.1 Evaluation of Panel Performance

Evaluating the competence of the panel is a crucial step in sensory analysis, as the consistency of the panel directly impacts the accuracy and reliability of the experimental data (Lefebvre et al. 2010). The competence of Panel 2 was assessed using the results from the QDA's 5‐point sensory evaluation.

**FIGURE 4:**
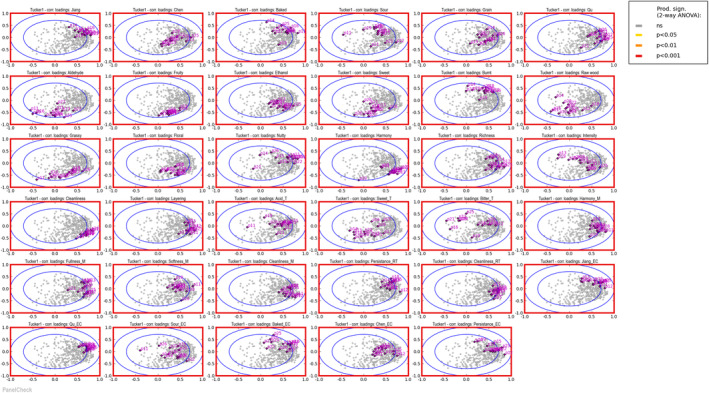
Consistency of the panel.

Tucker‐1 correlation loadings plots (Figure 4) were used to identify descriptors with potential performance issues (Yan, Chen, et al. 2021). A higher concentration of points for a given descriptor in the plot indicates greater consistency among panelists for that descriptor (Yu et al. 2022). All 35 sensory descriptors showed significant differences at the 0.001 level (red outer line). Among the 28 Jiang‐flavor Baijiu sensory attributes, excluding Bakes, Sour, Aldehyde, Raw wood, Intensity, Bitter_T, and Sour_EC, 13 tasters demonstrated relatively strong performance, indicating a high degree of consistency within the panel. The moderate consistency observed for these 7 sensory indicators suggests that they may be more challenging for tasters to understand, apply, and perceive in JFB. Additionally, tasters p21 in Qu and Nutty, p19 in Harmony, and p11 in Acid_T were notably distant from other panelists. These findings suggest that further training is necessary for these sensory indicators to ensure the quality of future JFB tastings.

#### 3.2.2 HCA and PCA of the QDA Results

**FIGURE 5:**
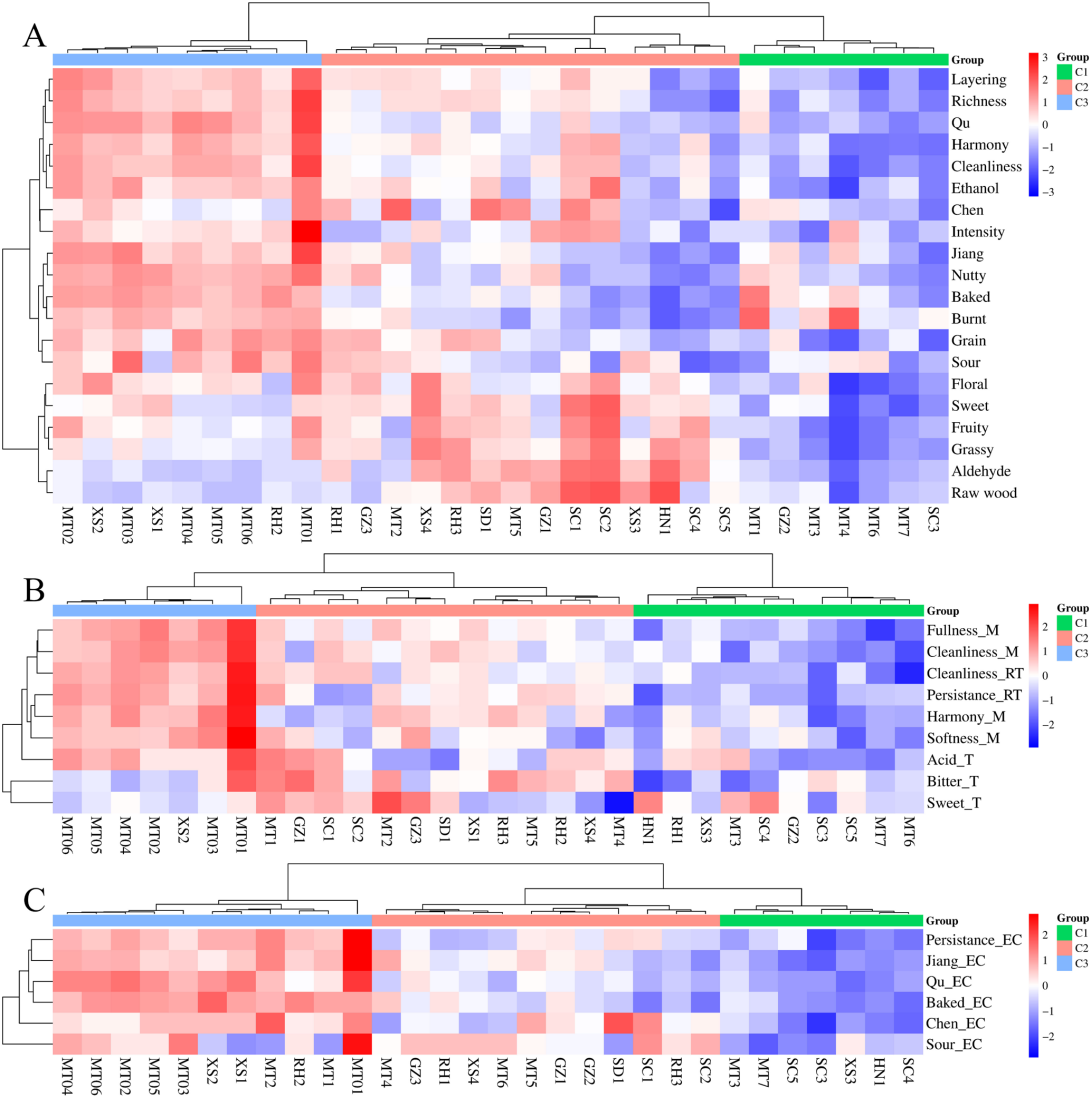
Cluster analysis of QDA data for sensory characteristics of 30 JFB samples. (A) Aroma, (B) taste and mouthfeel, (C) aroma in empty cup.

**FIGURE 6:**
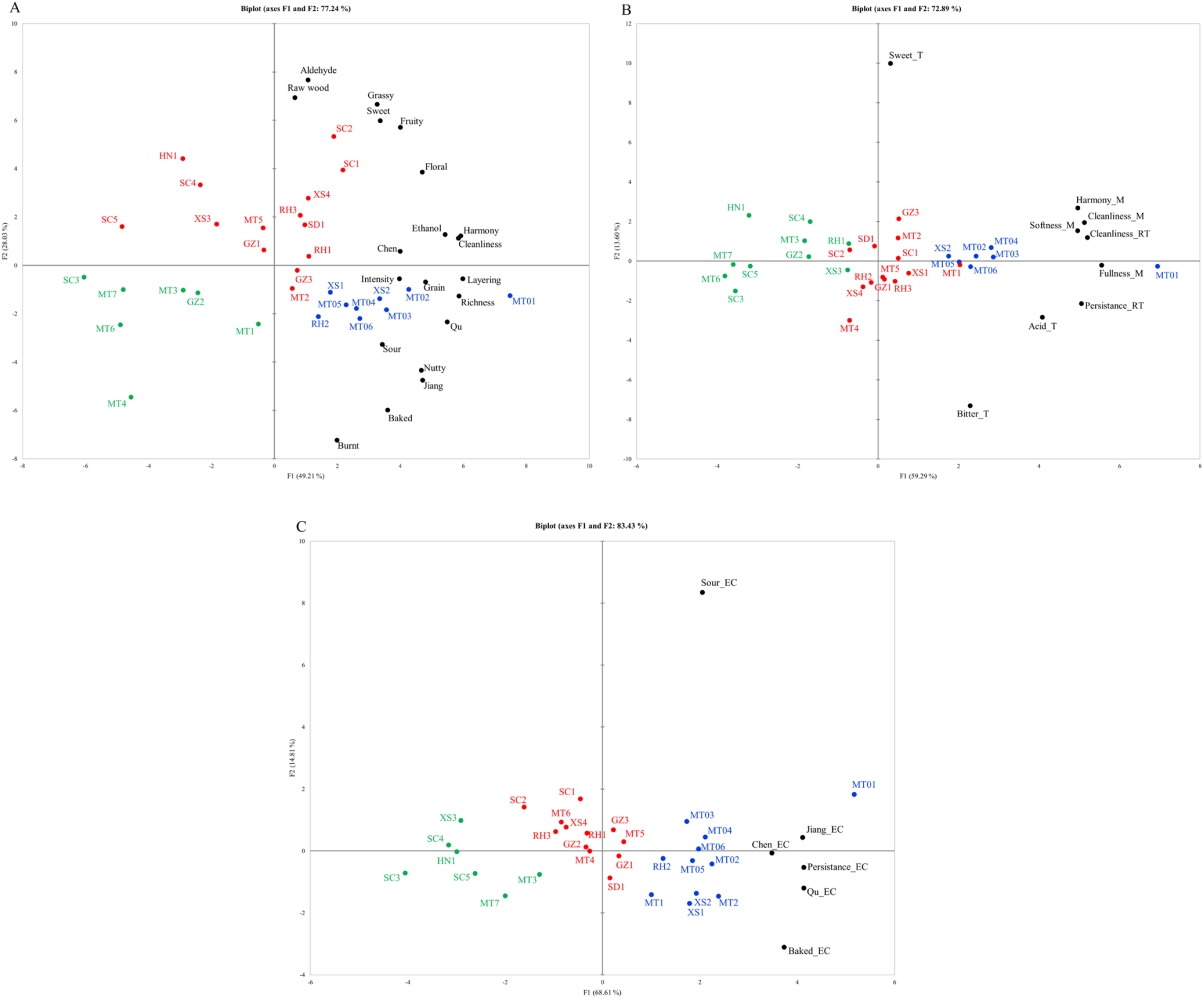
Principal component analysis of QDA data for sensory characteristics of 30 JFB samples. (A) Aroma, (B) taste and mouthfeel, (C) aroma in empty cup.

Samples, the panel, and descriptors are three crucial components of sensory analysis. In the present study, JFB sensory descriptors were first screened using multivariate statistical methods based on the panel's evaluation of the samples. Next, panel consistency was assessed using the tasting data from the samples and their corresponding sensory descriptors. The sensory characteristics of the samples were then analyzed by assigning points to the sensory indicators. The QDA results were clustered and analyzed across three dimensions: aroma, taste, and mouthfeel, as well as aroma in empty cup (Figures 5 and 6).

The sensory quality of each group of samples decreased progressively from C3 to C2 to C1, with C3 exhibiting the highest sensory quality and C1 the lowest. Specifically, in terms of aroma, C3 samples were distinguished by prominent Jiang, Qu, Cleanliness, and Layering attributes, while C2 samples were noted for Aldehyde and Raw wood, and C1 samples for Burnt. Regarding taste and mouthfeel, C3 samples excelled in Harmony_M, Fullness_M, Cleanliness_M, and Cleanliness_RT, whereas C2 samples stood out for Sweet_T. In terms of empty cup aroma, C3 samples excelled in Jiang_EC, Qu_EC, and Baked_EC. Overall, C3 samples led comprehensively across all three dimensions, C2 samples had some notable qualities in aroma, and C1 samples scored lower across the board.

The six samples from MTCQ1 were grouped together in each dimension, all displaying high sensory quality. This suggests that JFBs from different price points of MTCQ1 share strong consistency and stability in their distinctive styles, confirming to some extent the quality leadership of JFB from MTCQ1 across various price segments.

#### 3.2.3 Rader Plot Analysis

The QDA data were analyzed using one‐way ANOVA (Table S1), and radar plots were generated for six price segments and seven production regions. The price segments served as a basis for analyzing the sensory characteristics and quality of samples across different price points. The production regions were primarily examined to determine whether JFB from the same region shared common sensory characteristics and whether a unique blending method was used.

**FIGURE 7:**
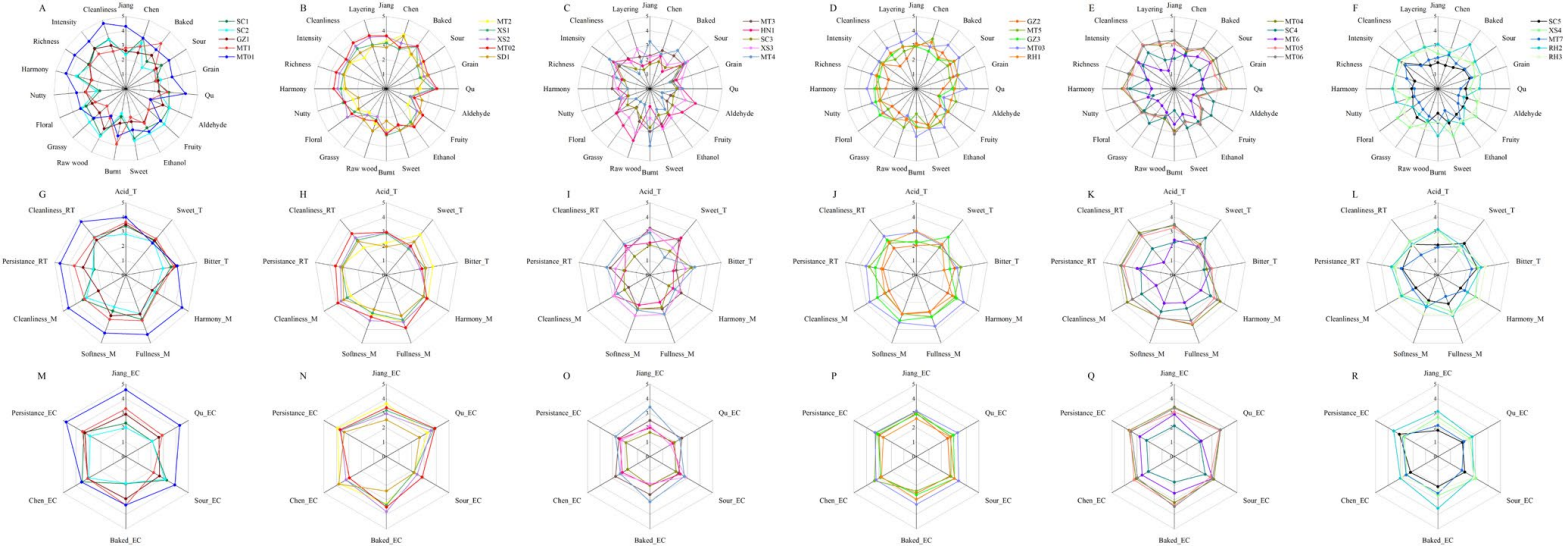
Radar plot analysis of QDA data for sensory characteristics of 30 Jiang‐flavor Baijiu samples (based on price grouping). (A–F) aroma dimensions, (G–L) taste and mouthfeel dimensions, (M–R) empty cup aroma dimensions; (A, G, M) H1 samples, (B, H, N) H2 samples, (C, I, O) M1 samples, (D, J, P) M2 samples, (E, K, Q) L1 samples, and (F, L, R) L2 samples.

As shown in Figure 7, MT01 had a higher overall profile in the aroma dimensions (Jiang 4.31, Grain 3.31, Qu 4.15, Nutty 3.42, Harmony 4.27, Richness 4.23, Intensity 4.15, Cleanliness 4.77, and Layering 4.19). However, its individual sensory attributes were not the most prominent in the sample set (e.g., MT1 Baked 3.96, SC2 Fruity 3.62, Ethanol 3.50, Sweet 3.65, Grassy 3.42, and MT03 Sour 3.65). When analyzing the price segments, the remaining four H1 samples compared to the five H2 samples showed that the latter had a more balanced and similar sensory profile. Among the five samples from M1 and M2, the M2 samples had a fuller and more consistent sensory profile, while the M1 samples exhibited a more variable sensory profile, with individual samples reaching the highest (e.g., HN1 Aldehyde 3.31, Raw Wood 3.77, MT4 Burnt 3.96) or the lowest scores (e.g., MT3 Intensity 2.62, HN1 Baked 1.46, Burnt 1.23, SC3 Jiang 1.69, Harmony 1.54, MT4 Grain 1.46, Aldehyde 0.88, Fruity 1.04, Ethanol 1.77, Sweet 1.50, Raw Wood 1.15, Grassy 1.12, Floral 1.19, Cleanliness 1.27). The sensory attributes of the 10 samples from L1 and L2 did not show wide fluctuations, and the sensory profiles of SC4, MT6, SC5, and MT7 were relatively low, with SC4 Sour at 2.15 and Nutty at 1.54, MT6 Layering at 1.31, SC5 Chen at 1.69 and Richness at 1.69, and MT7 Qu at 1.58 being the lowest scores for the corresponding attributes in the sample set. In terms of aroma, the study found that MT01 stood out significantly, with the sensory quality of JFB samples priced above RMB 1500 being unbalanced, those priced between RMB 700–1000 showing fluctuating sensory quality, and those priced below RMB 500 exhibiting both low and high quality.

MT01 exhibited a stronger overall profile in the taste and mouthfeel dimensions (Acid_T 4.00, Bitter_T 3.62, Harmony_M 4.50, Fullness_M 4.38, Softness_M 4.27, Cleanliness_M 4.58, Persistence_RT 4.62, Cleanliness_RT 4.81), though the Sweet_T attribute of MT2 was particularly notable with a score of 3.65. In terms of price segments, the remaining four H1 samples and the five H2 samples displayed a more stable and consistent sensory profile, which contrasts with the results of the aroma attributes. The sensory profiles of the five M1 samples were more compressed and volatile, with some individual samples receiving the lowest scores among the 30 JFBs for certain sensory attributes (e.g., HN1 Persistence_RT 1.75, SC3 Harmony_M 1.50, and MT4 Sweet_T 1.58), similar to the trends observed in the aroma attributes. In the L1 and L2 samples, the phenomenon of both low‐priced high quality and low‐priced low quality was evident. The high‐quality characteristics were primarily concentrated in the L1 samples, which exhibited sensory profiles similar to those of higher‐priced JFBs such as H2. Conversely, the lower quality was manifested by a contraction in the sensory profile and by individual attribute scores that were the lowest in the sample set, such as MT6 Cleanliness_M at 1.46, Cleanliness_RT at 1.15, SC5 Softness_M at 1.88, and MT7 Fullness_M at 1.58, reflecting similar trends to the aroma attributes. In the taste and mouthfeel dimensions, MT01 was found to be significantly ahead of the others, with samples above RMB 1000 exhibiting more balanced sensory qualities, those between RMB 700 and RMB 1000 showing more fluctuation in sensory qualities, and samples below RMB 500 displaying both low quality at low prices and high quality at low prices.

In the empty cup aroma dimension, MT01 continued to exhibit a stronger overall profile (Jiang_EC 4.62, Qu_EC 4.31, Sour_EC 3.92, and Persistence_EC 4.77), but the Baked_EC and Chen_EC attributes were most prominent in XS2 and SD1, both scoring 3.81. When considering each price segment, the balance and stability of sensory quality in the five H1 samples were weaker compared to the five H2 samples. SC1 and SC2, within the H1 group, had smaller sensory profiles, exemplifying the phenomenon of high price with low quality, while XS2 and SD1 in the H2 group had the most pronounced Baked_EC and Chen_EC attributes in the sample set. The sensory profiles of the M1 samples were significantly smaller than those of the M2 samples, indicating that the sensory quality of the mid‐price range samples (particularly RMB 700–1000) did not correspond to their labeled prices. For instance, SC3 had the lowest scores in Jiang_EC (1.67), Chen_EC (1.79), Persistence_EC (1.92), and XS3 in Qu_EC (1.63). Among the low‐priced samples, MT04, MT05, and MT06 exhibited fuller and more balanced sensory profiles, highlighting the presence of low‐price, high‐quality attributes.

**FIGURE 8:**
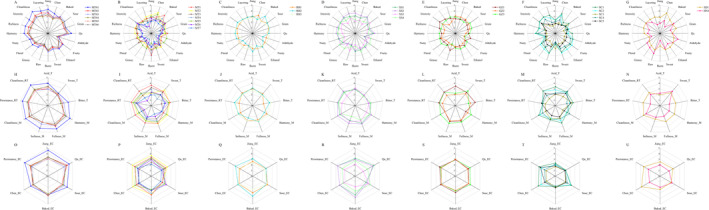
Radar plot analysis of QDA data for sensory characteristics of 30 Jiang‐flavor Baijiu samples (based on region grouping). (A–G) aroma dimensions, (H–N) taste and mouthfeel dimensions, (O–U) empty cup aroma dimensions; (A, H, O) MTCQ1 samples, (B, I, P) MTCQ2 samples, (C, J, Q) RSCQ samples, (D, K, R) XSCQ samples, (E, L, S) GZCQ samples, (F, M, T) SCCQ samples, (G, N, U), SDCQ and HNCQ samples.

From the production region radar plot (Figure 8), it is evident that all six samples of MTCQ1 exhibited highly similar sensory profiles across all sensory dimensions, with MT01 outperforming in most sensory attributes (e.g., Jiang, Qu, Cleanliness, etc.), which formed the outer edges of the plot. The remaining samples displayed comparable scores across all sensory attributes, showing mutual strengths and weaknesses. This likely indicates that the production region exerts a certain influence on the sensory quality of JFB. The sensory profiles of the XS1 and XS2 samples from XSCQ closely matched each other and were more similar to those of the MTCQ1 samples, consistent with the results of the cluster analysis. Similarly, the sensory profiles of the SC2 and SC1 samples from SCCQ were alike, likely due to the similarity in base wine blending methods. MTCQ2, which included seven samples—the largest number in this study—displayed a more condensed and highly diverse sensory profile, with each sample exhibiting distinct sensory characteristics. This reflects the varied nature of the MTCQ2 JFB market. In previous studies, flavor compounds of JFB were primarily used as key markers for identifying the production region. The sensory lexicon constructed in this study has shown some effectiveness in distinguishing the production regions of JFB (Huang et al. 2023), but further examination using sensory analysis of a larger number of samples is needed in the future.

## 4 Conclusion

Thirty samples of Jiang‐flavor Baijiu (JFB) were evaluated by two trained sensory panels. The initial list of 88 sensory descriptors for JFB was extensively researched, collected, and organized. Panel 1 conducted a sensory evaluation and screening of these descriptors, leading to the development of a refined sensory lexicon for JFB. This lexicon contains 35 descriptors (15 for aroma types, 5 for aroma features, 3 for taste, 4 for mouthfeel, 1 for residual taste, and 6 for empty cup aroma), along with their definitions and physical references, to describe the sensory attributes of JFB and guide tasters in their evaluations. These 35 descriptors were statistically significant at the 0.001 level and were better understood and applied by Panel 2. Clustering analysis identified Jiang, Qu, Cleanliness, Aldehyde, Raw Wood, Burnt, Harmony_M, Fullness_M, Cleanliness_M, Cleanliness_RT, Sweet, Jiang_EC, Qu_EC, and Baked_EC as key indicators for differentiating the sensory quality of various JFBs. The radar plot and one‐way ANOVA results indicated that several samples exhibited distinctive aroma characteristics, with MT01 showing the highest overall sensory quality. There were larger fluctuations in the sensory quality among samples priced between 700 and 1000 RMB, and some low‐priced samples below 500 RMB demonstrated unexpectedly high quality. The six samples from MTCQ1 and the XS1 and XS2 samples from XSCQ had closely matched sensory profiles, while SC2 and SC1 samples from SCCQ also showed similar profiles, likely due to the influence of production region conditions and the similarity in base wine blending methods. This study successfully established a preliminary sensory lexicon for JFB, combined with the QDA method, which proved to be accurate and applicable for the sensory evaluation of JFB. It also serves as a reference for developing sensory lexicons for other types of Baijiu in China. Future research should explore the intrinsic link between the sensory characteristics and flavor compounds of JFB to enhance the development of JFB with diverse sensory profiles.

## Author Contributions

**Junjie Niu:** data curation (lead), writing – original draft (lead). **Yubo Yang:** investigation (equal). **Lei Zhao:** supervision (equal). **Nian Cao:** investigation (equal), project administration (equal). **Xiaolin Xiong:** project administration (equal). **Zhenyu Yun:** supervision (equal). **Fan Yang:** investigation (equal), project administration (equal). **Yang Xu:** project administration (equal). **Huabin Tu:** investigation (lead). **Kui Zhong:** supervision (equal). **Houyin Wang:** supervision (equal). **Haiyan Gao:** supervision (lead), writing – review and editing (equal). **Yao Zhang:** project administration (equal). **Zhen Qin:** supervision (equal), writing – review and editing (equal). **Li Wang:** investigation (lead), project administration (lead). **Bolin Shi:** supervision (equal).

## Conflicts of Interest

The authors declare no conflicts of interest.

## Supporting information


**FIGURE S1.** ANOVA of QDA data for sensory characteristics of 30 JFB samples.

**TABLE S1.** ANOVA of QDA data for sensory characteristics of 30 JFB samples.

## Data Availability

The data that support the findings of this study are available on request from the corresponding author.

## References

[fsn34652-bib-0001] Chambers, E., IV , K. Sanchez , U. X. T. Phan , R. Miller , G. V. Civille , and B. Di Donfrancesco . 2016. “Development of a “Living” Lexicon for Descriptive Sensory Analysis of Brewed Coffee.” Journal of Sensory Studies 31, no. 6: 465–480. 10.1111/joss.12237.

[fsn34652-bib-0002] Chen, Y. P. , and H. Y. Chung . 2016. “Development of A Lexicon for Commercial Plain Sufu (Fermented Soybean Curd).” Journal of Sensory Studies 31, no. 1: 22–33. 10.1111/joss.12187.

[fsn34652-bib-0003] De Pelsmaeker, S. , G. De Clercq , X. Gellynck , and J. J. Schouteten . 2019. “Development of a Sensory Wheel and Lexicon for Chocolate.” Food Research International 116: 1183–1191. 10.1016/j.foodres.2018.09.063. 30716904

[fsn34652-bib-0004] García‐Gómez, B. , N. Fernández‐Canto , M. L. Vázquez‐Odériz , M. Quiroga‐García , N. Muñoz‐Ferreiro , and M. Á. Romero‐Rodríguez . 2022. “Sensory Descriptive Analysis and Hedonic Consumer Test for Galician Type Breads.” Food Control 134: 108765. 10.1016/j.foodcont.2021.108765.

[fsn34652-bib-0005] GB/T 10345‐2022 . 2017. “Method of Analysis for Baijiu.”

[fsn34652-bib-0006] GB/T 16861‐1997 . 1997. “Sensory Analysis‐Identification and Selection of Descriptors for Establishing a Sensory Profile by a Multidimensional Approach.”

[fsn34652-bib-0007] GB/T 33405‐2016 . 2016. “Terminology of Baijiu Sensory Evaluation.”

[fsn34652-bib-0008] Ge, J. , Y. Qi , W. Yao , et al. 2023. “Identification of Trace Components in Sauce‐Flavor Baijiu by High‐Resolution Mass Spectrometry.” Molecules 28: 273. 10.3390/molecules28031273. PMC9920578 36770938

[fsn34652-bib-0009] Gulzar, N. , A. Sameen , R. Muhammad Aadil , et al. 2020. “Descriptive Sensory Analysis of Pizza Cheese Made From Mozzarella and Semi‐Ripened Cheddar Cheese Under Microwave and Conventional Cooking.” Food 9, no. 2: 214. 10.3390/foods9020214. PMC7073562 32092858

[fsn34652-bib-0010] Huang, H. , Y. Wu , H. Chen , et al. 2023. “Identification of Regionalmarkers Based on the Flavor Molecular Matrix Analysis of Sauce‐Aroma Style Baijiu.” Journal of the Science of Food and Agriculture 103, no. 15: 7434–7444. 10.1002/jsfa.12823. 37395138

[fsn34652-bib-0011] Lawless, L. J. R. , and G. V. Civille . 2013. “Developing Lexicons: A Review.” Journal of Sensory Studies 28, no. 4: 270–281. 10.1111/joss.12050.

[fsn34652-bib-0012] Lefebvre, A. , J. F. Bassereau , A. M. Pensé‐Lheritier , C. Rivère , N. Harris , and R. Duchamp . 2010. “Recruitment and Training of a Sensory Expert Panel to Measure the Touch of Beverage Packages: Issue and Methods Employed.” Food Quality and Preference 21, no. 1: 156–164. 10.1016/j.foodqual.2009.08.020.

[fsn34652-bib-0013] Li, H. , X. Zhang , X. Gao , et al. 2023. “Comparison of the Aroma‐Active Compounds and Sensory Characteristics of Different Grades of Light‐Flavor Baijiu.” Food 12, no. 6: 12. 10.3390/foods12061238. PMC10048497 36981164

[fsn34652-bib-0014] Li, J. , Q. Zhang , and B. Sun . 2023. “Chinese Baijiu and Whisky: Research Reservoirs for Flavor and Functional Food.” Food 12, no. 15: 15. 10.3390/foods12152841. PMC10417287 37569110

[fsn34652-bib-0015] Li, T. , J. Wang , B. Xu , et al. 2023. “Comparative Analysis of the Differences Among Langya Flavor Baijiu and Strong and Soy Sauce Flavor Baijiu by Targeted Flavor Analysis.” Journal of Food Composition and Analysis 122: 105479. 10.1016/j.jfca.2023.105479.

[fsn34652-bib-0016] Niu, J. , B. Shi , H. Wang , et al. 2024. “Formation, Development and Trend of Sensory Quality Description of Jiang‐Flavor Baijiu.” Food Science 45, no. 5: 324–334. 10.7506/spkx1002-6630-20230703-019.

[fsn34652-bib-0017] Niu, J. , B. Shi , Y. Yang , et al. 2023. “Baijiu Tasting Under the Perspective of Modern Sensory Sciences: Case Study on Jiang‐Flavor Baijiu.” Proceedings of the 6th International Symposium on Baijiu Technology: 165–187.

[fsn34652-bib-0018] Niu, Y. , W. Zhao , Z. Xiao , J. Zhu , W. Xiong , and F. Chen . 2023. “Characterization of Aroma Compounds and Effects of Amino Acids on the Release of Esters in Laimao Baijiu.” Journal of the Science of Food and Agriculture 103, no. 4: 1784–1799. 10.1002/jsfa.12281. 36260337

[fsn34652-bib-0028] Qin, D. , Z. Wu , Y. Shen , et al. 2023. “Characterization of Empty Cup Aroma in Soy Sauce Aroma Type Baijiu by Vacuum Assisted Sorbent Extraction.” Journal of Food Composition and Analysis 117: 105147. 10.1016/j.jfca.2023.105147.

[fsn34652-bib-0019] Qin, D. , S. Lv , Y. Shen , et al. 2024. “Decoding the Key Compounds Responsible for the Empty Cup Aroma of Soy Sauce Aroma Type Baijiu.” Food Chemistry 434: 137466. 10.1016/j.foodchem.2023.137466. 37741247

[fsn34652-bib-0020] Solomando, J. C. , T. Antequera , S. Ventanas , and T. Perez‐Palacios . 2021. “Sensory Profile and Consumer Perception of Meat Products Enriched With EPA and DHA Using Fish Oil Microcapsules.” International Journal of Food Science & Technology 56, no. 6: 2926–2937. 10.1111/ijfs.14932.

[fsn34652-bib-0021] Souza Gonzaga, L. , D. L. Capone , S. E. P. Bastian , L. Danner , and D. W. Jeffery . 2019. “Using Content Analysis to Characterise the Sensory Typicity and Quality Judgements of Australian Cabernet Sauvignon Wines.” Food 8, no. 12: 691. 10.3390/foods8120691. PMC6963444 31861236

[fsn34652-bib-0022] Sun, Y. , Y. Ma , S. Chen , Y. Xu , and K. Tang . 2021. “Exploring the Mystery of the Sweetness of Baijiu by Sensory Evaluation, Compositional Analysis and Multivariate Data Analysis.” Food 10, no. 11: 2843. 10.3390/foods10112843. PMC8622430 34829124

[fsn34652-bib-0023] Wang, L. , S. Fan , Y. Yan , L. Yang , S. Chen , and Y. Xu . 2020. “Characterization of Potent Odorants Causing a Pickle‐Like Off‐Odor in Moutai‐Aroma Type Baijiu by Comparative Aroma Extract Dilution Analysis, Quantitative Measurements, Aroma Addition, and Omission Studies.” Journal of Agricultural and Food Chemistry 68, no. 6: 1666–1677. 10.1021/acs.jafc.9b07238. 31957444

[fsn34652-bib-0024] Wang, L. , L. Zhu , F. Zheng , et al. 2021. “Determination and Comparison of Flavor (Retronasal) Threshold Values of 19 Flavor Compounds in Baijiu.” Journal of Food Science 86, no. 5: 2061–2074. 10.1111/1750-3841.15718. 33884627

[fsn34652-bib-0025] Wang, Y.‐H. , Y.‐Y. Yang , F. Xu , Q.‐D. Zhang , X.‐K. Wang , and H. Xu . 2023. “Lexicon Development and Quantitative Descriptive Analysis of Chinese Steamed Bread.” Journal of Cereal Science 111: 103654. 10.1016/j.jcs.2023.103654.

[fsn34652-bib-0026] Wu, J. , R. Chen , X. Li , et al. 2023. “Comprehensive Identification of Key Compounds in Different Quality Grades of Soy Sauce‐Aroma Type Baijiu by HS‐SPME‐GC‐MS Coupled With Electronic Nose.” Frontiers in Nutrition 10: 2527. 10.3389/fnut.2023.1132527. PMC10028209 36960200

[fsn34652-bib-0027] Wu, Y. , Y. Hou , H. Chen , et al. 2022. “‘Key Factor’ for Baijiu Quality: Research Progress on Acid Substances in Baijiu.” Food 11: 959. 10.3390/foods11192959. PMC9562207 36230035

[fsn34652-bib-0029] Xue, W. , L. Jian , W. Qian , et al. 2023. “Research Progress on the Effect of Bacillus on Flavor Substances of Maotai Flavor Baijiu.” Food Science and Technology 43: e101422. 10.1590/fst.101422.

[fsn34652-bib-0030] Yan, Q. , K. Zhang , W. Zou , and Y. Hou . 2021. “Three Main Flavour Types of Chinese Baijiu: Characteristics, Research, and Perspectives.” Journal of the Institute of Brewing 127, no. 4: 317–326. 10.1002/jib.669.

[fsn34652-bib-0031] Yan, Y. , S. Chen , Y. Nie , and Y. Xu . 2021. “Quantitative Analysis of Pyrazines and Their Perceptual Interactions in Soy Sauce Aroma Type Baijiu.” Food 10: 441. 10.3390/foods10020441. PMC7922815 33671408

[fsn34652-bib-0032] Yang, Y. , J. Niu , B. Shi , et al. 2024. “Study on the Differentiation of Sensory Quality of Mainstream Jiang‐Flavor Baijiu in the Chinese Market Based on Pivot Profile.” Journal of Food Science 89: 7958–7975. 10.1111/1750-3841.17383. 39363247

[fsn34652-bib-0033] Yu, M. , C. Zheng , Q. Xie , et al. 2022. “Flavor Wheel Construction and Sensory Profile Description of Human Milk.” Nutrients 14, no. 24: 387. 10.3390/nu14245387. 36558546 PMC9783944

