# Supplementary material for: Sensory Lexicon Construction and Quantitative Descriptive Analysis of Jiang‐Flavor Baijiu
Source: Food Sci Nutr. 2025 Feb 18;13(2):e4652. doi: 10.1002/fsn3.4652 (PMC11833230; doi:10.1002/fsn3.4652)
Supplement: Supplementary file 2 — TABLE S1. ANOVA of QDA data for sensory characteristics of 30 JFB samples. [file FSN3-13-e4652-s001.zip › fsn34652-sup-0002-TableS1.docx]

Table S1 ANOVA of QDA data for sensory characteristics of 30 JFB samples.
